# Supplementary material for: The dual coding of a single sex pheromone receptor in Asian honeybee Apis cerana
Source: Commun Biol. 2024 Apr 25;7:502. doi: 10.1038/s42003-024-06206-5 (PMC11045764; doi:10.1038/s42003-024-06206-5)
Supplement: Supplementary file 2 — Supplementary Information [file 42003_2024_6206_MOESM2_ESM.pdf]

Supplementary Materials for

The dual coding of a single sex pheromone receptor in Asian Honeybee *Apis cerana*

Haoqin Ke *et al.*

*Corresponding author. Email: Dr. Bingzhong Ren (bzren@nenu.edu.cn), Dr. Yinliang Wang (wangyl392@nenu.edu.cn)

This PDF file includes:

Supplementary Table 1 to Table 3
Supplementary Fig. 1 to Fig. 7

Supplementary Table 1 Quantitative GC-MS analysis of 9-ODA and HOB (N = 3).

**Caste**	**9-ODA**	**HOB**
Virgin queen	15.951 ± 6.520 μg	-
Mated queen	35.993 ± 3.560 μg	8.019 ± 1.589 μg
Drone	-	-
Worker	-	-

Supplementary Table 2 Primers list in this study.

Purpose	Primer	Primer (5’ - 3’)
Gene cloning	Orco-F	ACGTACCCGCCTCAA
	Orco-R	TCATTTAGTCACAGTTTCCA
	Or11-F	TCACGAACAAGCTTTCATCGG
	Or11-R	GAAAGTGAACAAAGTGCTGTGTACA
Infusion cloning	Orco-F	AATTCCCCGGGGATCCACCATGATGAAGTTCAAGCAACAGGG
	Orco-R	TAACCAGATCAAGCTTTCACTTCAGTTGCACCAACACC
	Or11-F	AATTCCCCGGGGATCCACCATGGTCCAAATTAGAAACGCGAAAG
	Or11-R	TAACCAGATCAAGCTTTTACGTAACTGTACGTAACATATTC
qPCR	Orco-F	GGATCAGAGGAGGCCAAAAC
	Orco-R	CCAACACCGAAGCAAAGAGA
	Or11-F	ATGTGCGGTTTGCTGAAGA
	Or11-R	CGAGAAGGTGCCAATGACG
	β-actin-F	GGCTCCCGAAGAACATCC
	β-actin-R	TGCGAAACACCGTCACCC
In situ hybridization	Or11-F	AATTCCCCGGGGATCCACCTTACGTAACTGTACGTAACATATTC
	Or11-R	TAACCAGATCAAGCTTATGGTCCAAATTAGAAACGCGAAAG

Note: The underlined DNA sequence was the enzyme restriction site.

Supplementary Table 3 Compounds used for deorphanize the *AcerOr11*.

Compound	Cas No.	Source	Company	Country	Purity	Structure
(*E*)-9-Oxo-2-decenoic acid (9-ODA)	334-20-3	Queen retinue pheromone	Chem-strong Biotechnology Co., LTD	Shenzhen, China	95.0 %	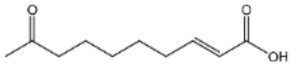
9-Hydroxy-2-decenoic acid (9-HDA)	4448-33-3	Queen retinue pheromone	Albs Biotechnology Co., LTD	Changsha, China	95.0 %	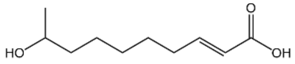
Methyl *p*-hydroxybenzoate (HOB)	99-76-3	Queen retinue pheromone	Rhawn Biotechnology Co., LTD	Shanghai, China	99.0 %	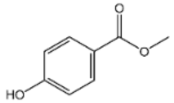
4-Hydroxy-3-methoxyphenylethanol (HVA)	2380-78-1	Queen retinue pheromone	Rhawn Biotechnology Co., LTD	Shanghai, China	98.0 %	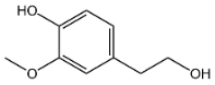
(E)-10-Hydroxy-dec-2-enoic acid (10-HDA)	765-01-5	Queen retinue pheromone	Rhawn Biotechnology Co., LTD	Shanghai, China	98.0 %	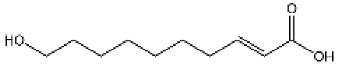
10-Hydroxy-decanoic acid (10-HDAA)	1679-53-4	Queen retinue pheromone	Rhawn Biotechnology Co., LTD	Shanghai, China	96.0 %	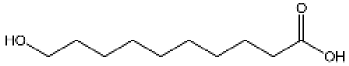
Methyl oleate (MO)	112-62-9	Queen retinue pheromone	Aladdin Biochemical Technology Co., LTD	Shanghai, China	99.0 %	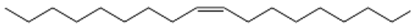
1-Hexadecanol (PA)	36653-82-4	Queen retinue pheromone	Aladdin Biochemical Technology Co., LTD	Shanghai, China	99.0 %	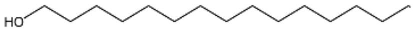
Linolenic acid (LA)	463-40-1	Queen retinue pheromone	Merda Technology Co., LTD	Beijing, China	99.0 %	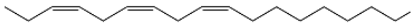
Geraniol	106-24-1	Nasonov pheromone	Sigma-Aldrich LLC.	MO, USA	98.0 %	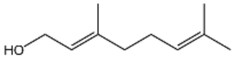
Geranial	5392-40-5	Nasonov pheromone	Aladdin Biochemical Technology Co., LTD	Shanghai, China	97.0 %	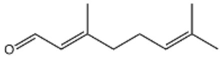

Compound	Cas No.	Source	Company	Country	Purity	Structure
Farnesol	4602-84-0	Nasonov pheromone	Bidepharm Medical Technology Co., LTD	Shanghai, China	95.0 %	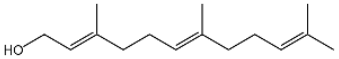
Nerol	106-25-2	Nasonov pheromone	Rhawn Biotechnology Co., LTD	Shanghai, China	97.0 %	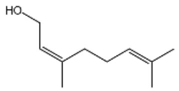
Geranic acid	459-80-3	Nasonov pheromone	Rhawn Biotechnology Co., LTD	Shanghai, China	90.0 %	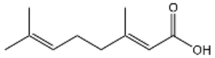
(E)-Citral	141-27-5	Nasonov pheromone	Kaiwei Biotechnology Co., LTD	Liaoning, China	95.0 %	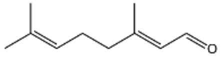
Benzyl acetate	140-11-4	Alarm pheromone	Rhawn Biotechnology Co., LTD	Shanghai, China	99.0 %	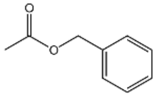
Isoamyl acetate	123-92-2	Alarm pheromone	Aladdin Biochemical Technology Co., LTD	Shanghai, China	99.0 %	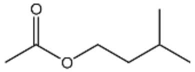
Butyl acetate	123-86-4	Alarm pheromone	Aladdin Biochemical Technology Co., LTD	Shanghai, China	99.7 %	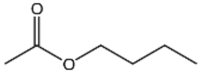
Hexyl acetate	142-92-7	Alarm pheromone	Sigma-Aldrich LLC.	MO, USA	99.0 %	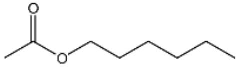
Octyl acetate	112-14-1	Alarm pheromone	Rhawn Biotechnology Co., LTD	Shanghai, China	98.0 %	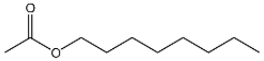
Decyl acetate	112-17-4	Alarm pheromone	Aladdin Biochemical Technology Co., LTD	Shanghai, China	98.0 %	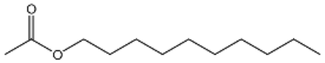
Phenyl acetate	122-79-2	Alarm pheromone	Aladdin Biochemical Technology Co., LTD	Shanghai, China	98.0 %	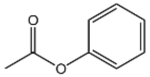

Compound	Cas No.	Source	Company	Country	Purity	Structure
Octanoic acid	124-07-2	Alarm pheromone	Aladdin Biochemical Technology Co., LTD	Shanghai, China	98.0 %	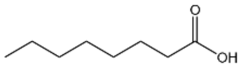
Benzoic acid	65-85-0	Alarm pheromone	Aladdin Biochemical Technology Co., LTD	Shanghai, China	99.5 %	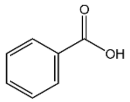
Phenol	108-95-2	Alarm pheromone	Aladdin Biochemical Technology Co., LTD	Shanghai, China	99.0 %	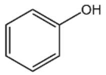
p-Cresol	106-44-5	Alarm pheromone	Aladdin Biochemical Technology Co., LTD	Shanghai, China	99.0 %	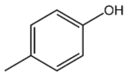
1-Hexanol	111-27-3	Alarm pheromone	Aladdin Biochemical Technology Co., LTD	Shanghai, China	95.0 %	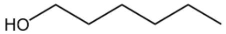
1-Butanol	71-36-3	Alarm pheromone	Rhawn Biotechnology Co., LTD	Shanghai, China	99.0 %	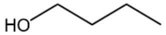
1-Pentanol	71-41-0	Alarm pheromone	Aladdin Biochemical Technology Co., LTD	Shanghai, China	99.5 %	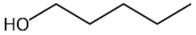
3-Methyl-1-butanol	123-51-3	Alarm pheromone	Rhawn Biotechnology Co., LTD	Shanghai, China	98.0 %	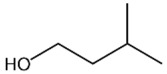
1-Octanol	111-87-5	Alarm pheromone	Aladdin Biochemical Technology Co., LTD	Shanghai, China	99.5 %	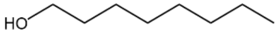
1-Octadecanol	112-92-5	Alarm pheromone	Rhawn Biotechnology Co., LTD	Shanghai, China	99.0 %	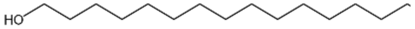
1-Eicosanol	629-96-9	Alarm pheromone	Bidepharm Medical Technology Co., LTD	Shanghai, China	98.0 %	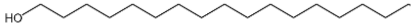

Compound	Cas No.	Source	Company	Country	Purity	Structure
2-Heptanone	110-43-0	Alarm pheromone	Sigma-Aldrich LLC.	MO, USA	99.0 %	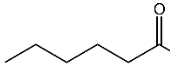
trans-2-Octen-1-ol	18409-17-1	Alarm pheromone	Sigma-Aldrich LLC.	MO, USA	97.0 %	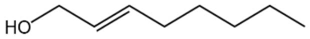
2-Nonanol	628-99-9	Alarm pheromone	Rhawn Biotechnology Co., LTD	Shanghai, China	98.0 %	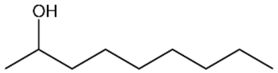
2-Aminoacetophenone	551-93-9	Queen feces pheromone	Bidepharm Medical Technology Co., LTD	Shanghai, China	99.5 %	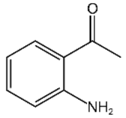
Decyl decanoate	1654-86-0	Tergum pheromone	Bidepharm Medical Technology Co., LTD	Shanghai, China	98.0 %	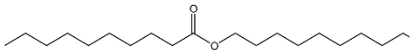
Methyl stearate	112-61-8	Brood pheromone	Aladdin Biochemical Technology Co., LTD	Shanghai, China	99.0 %	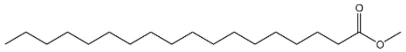
Ethyl stearate	111-61-5	Brood pheromone	Macklin Biochemical Technology Co., LTD	Shanghai, China	99.0 %	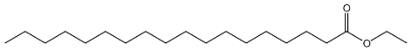
Methyl palmitate	112-39-0	Brood pheromone	Aladdin Biochemical Technology Co., LTD	Shanghai, China	99.0 %	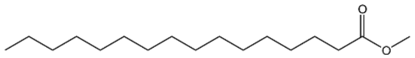
Ethyl palmitate	628-97-7	Brood pheromone	Aladdin Biochemical Technology Co., LTD	Shanghai, China	98.0 %	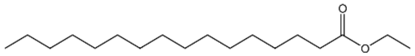
Ethyl oleate	111-62-6	Brood pheromone	BioDuly Biotechnology LTD	Nanjing, China	98.0 %	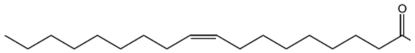
Methyl linoleate	112-63-0	Brood pheromone	Bidepharm Medical Technology Co., LTD	Shanghai, China	100.0 %	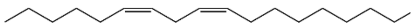
Ethyl linoleate	544-35-4	Brood pheromone	Rhawn Biotechnology Co., LTD	Shanghai, China	97.0 %	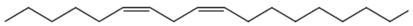

Compound	Cas No.	Source	Company	Country	Purity	Structure
β-Ocimene	13877-91-3	Brood pheromone	Sigma-Aldrich LLC.	MO, USA	90.0%	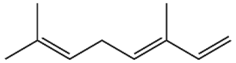
Myrcene	123-35-3	Plant volatiles	Rhawn Biotechnology Co., LTD	Shanghai, China	90.0%	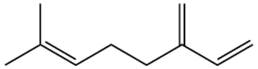
3-Carene	13466-78-9	Plant volatiles	TCI Development Co., LTD	Shanghai, China	90.0%	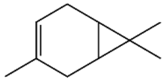
DL-Menthol	1490-04-6	Plant volatiles	Aladdin Biochemical Technology Co., LTD	Shanghai, China	98.0%	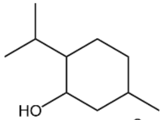
β-Ionone	14901-07-6	Plant volatiles	Sigma-Aldrich LLC.	MO, USA	96.0%	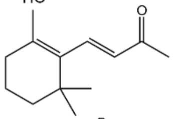
Methyl salicylate	119-36-8	Plant volatiles	JK scientific	Beijing, China	99.0%	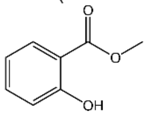
3,4-Dimethylbenzaldehyde	5973-71-7	Plant volatiles	Aladdin Biochemical Technology Co., LTD	Shanghai, China	97.0%	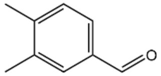
β-Citronellol	106-22-9	Plant volatiles	Sigma-Aldrich LLC.	MO, USA	95.0%	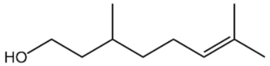
1,8-Cineole	470-82-6	Plant volatiles	Aladdin Biochemical Technology Co., LTD	Shanghai, China	99.5%	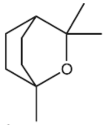
Phenylacetaldehyde	122-78-1	Plant volatiles	Sigma-Aldrich LLC.	MO, USA	90.0%	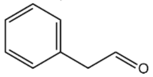
2-Isobutyl-3-methoxypyrazine	24683-00-9	Plant volatiles	Aladdin Biochemical Technology Co., LTD	Shanghai, China	99.0%	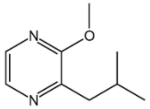

Compound	Cas No.	Source	Company	Country	Purity	Structure
Dodecanoic acid	143-07-7	Plant volatiles	Aladdin Biochemical Technology Co., LTD	Shanghai, China	98.0 %	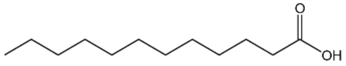
Tetradecanoic acid	544-63-8	Plant volatiles	Aladdin Biochemical Technology Co., LTD	Shanghai, China	98.0 %	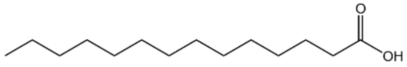
Hexadecanoic acid	57-10-3	Plant volatiles	Aladdin Biochemical Technology Co., LTD	Shanghai, China	98.0 %	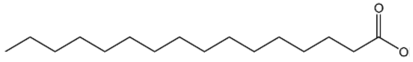
Oleic acid	112-80-1	Plant volatiles	Aladdin Biochemical Technology Co., LTD	Shanghai, China	98.0 %	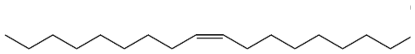
Linoleic acid	60-33-3	Plant volatiles	Aladdin Biochemical Technology Co., LTD	Shanghai, China	95.0 %	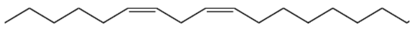
Stearic acid	57-11-4	Plant volatiles	Acmec Biochemical Technology Co., LTD	Shanghai, China	98.0 %	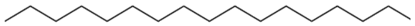
Methyl myristate	124-10-7	Plant volatiles	Aladdin Biochemical Technology Co., LTD	Shanghai, China	98.0 %	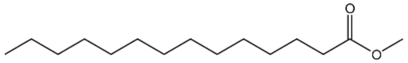
Dodecanal	112-54-9	Plant volatiles	Aladdin Biochemical Technology Co., LTD	Shanghai, China	95.0 %	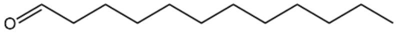
Octadecanal	638-66-4	Plant volatiles	Aladdin Biochemical Technology Co., LTD	Shanghai, China	97.0 %	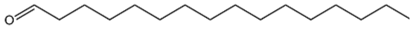
Oleamide	301-02-0	Plant volatiles	Bidepharm Medical Technology Co., LTD	Shanghai, China	98.0 %	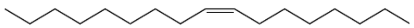
Linalool	78-70-6	Plant volatiles	Sigma-Aldrich LLC.	MO, USA	97.0 %	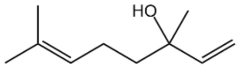

Compound	Cas No.	Source	Company	Country	Purity	Structure
3,7-Dimethyl-1-octanol	106-21-8	Plant volatiles	Bidepharm Medical Technology Co., LTD	Shanghai, China	98.0 %	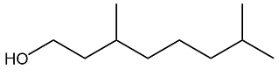
Citronellal	106-23-0	Plant volatiles	Heowns Chemical TecHhnology Co., LTD	Wuhan, China	96.0 %	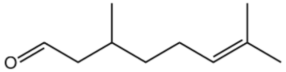
Eugenol	97-53-0	Plant volatiles	Sigma-Aldrich LLC.	MO, USA	99.0 %	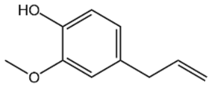
Geranyl acetate	105-87-3	Plant volatiles	Bidepharm Medical Technology Co., LTD	Shanghai, China	95.0 %	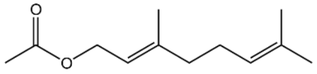
α-Methylcinnamaldehyde	101-39-3	Plant volatiles	Aladdin Biochemical Technology Co., LTD	Shanghai, China	95.0 %	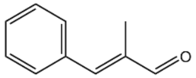
Benzaldehyde	100-52-7	Plant volatiles	Aladdin Biochemical Technology Co., LTD	Shanghai, China	99.5 %	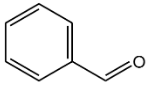
Octanal	124-13-0	Plant volatiles	Sigma-Aldrich LLC.	MO, USA	99.0 %	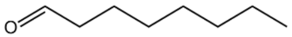
Cis-jasmone	488-10-8	Plant volatiles	Aladdin Biochemical Technology Co., LTD	Shanghai, China	98.0 %	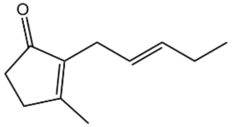
Indole	120-72-9	Plant volatiles	Aladdin Biochemical Technology Co., LTD	Shanghai, China	99.0 %	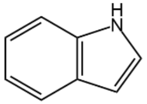
1- Nonanol	143-08-8	Plant volatiles	Bidepharm Medical Technology Co., LTD	Shanghai, China	98.0 %	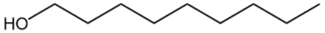
1-Octen-3-ol	3391-86-4	Plant volatiles	Aladdin Biochemical Technology Co., LTD	Shanghai, China	98.0 %	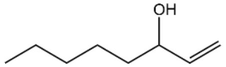

Compound	Cas No.	Source	Company	Country	Purity	Structure
β-Caryophllene	87-44-5	Plant volatiles	Bidepharm Medical Technology Co., LTD	Shanghai, China	90.0 %	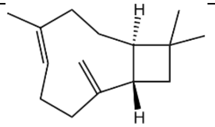
Ethyl acetate	141-78-6	Plant volatiles	Aladdin Biochemical Technology Co., LTD	Shanghai, China	99.5 %	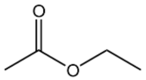
Piperitone	89-81-6	Plant volatiles	Macklin Biochemical Technology Co., LTD	Shanghai, China	98.0 %	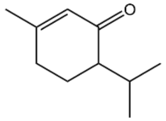
Nonanal	124-19-6	Plant volatiles	Sigma-Aldrich LLC.	MO, USA	95.0 %	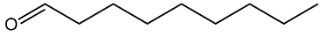
Ethyl cinnamate	103-36-6	Plant volatiles	Aladdin Biochemical Technology Co., LTD	Shanghai, China	99.0 %	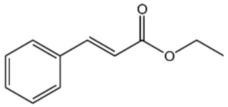
β-Pinene	18172-67-3	Plant volatiles	Rhawn Biotechnology Co., LTD	Shanghai, China	98.0 %	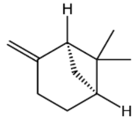
Trans-2-Hexenal	6728-26-3	Plant volatiles	Bidepharm Medical Technology Co., LTD	Shanghai, China	98.0 %	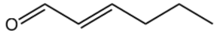
Benzyl alcohol	100-51-6	Plant volatiles	Rhawn Biotechnology Co., LTD	Shanghai, China	99.0 %	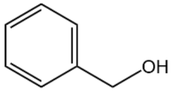
2-Phenylethanol	60-12-8	Plant volatiles	Aladdin Biochemical Technology Co., LTD	Shanghai, China	99.0 %	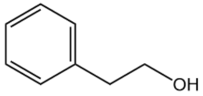
Phenethyl acetate	103-45-7	Plant volatiles	Rhawn Biotechnology Co., LTD	Shanghai, China	99.0 %	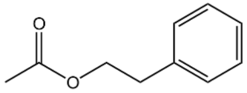
cis-3-Hexenyl acetate	3681-71-8	Plant volatiles	Rhawn Biotechnology Co., LTD	Shanghai, China	98.0 %	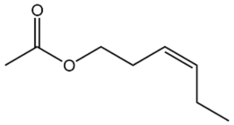

Compound	Cas No.	Source	Company	Country	Purity	Structure
α-Caryophyllene	6753-98-6	Plant volatiles	Aladdin Biochemical Technology Co., LTD	Shanghai, China	99.0 %	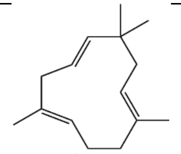
Limonene	138-86-3	Plant volatiles	Rhawn Biotechnology Co., LTD	Shanghai, China	95.0 %	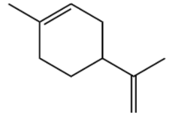
Hexanal	66-25-1	Plant volatiles	Aladdin Biochemical Technology Co., LTD	Shanghai, China	99.0 %	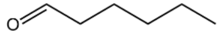
Dihydro-ionone	17283-81-7	Plant volatiles	Rhawn Biotechnology Co., LTD	Shanghai, China	98.0 %	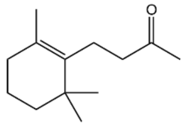
3-Octanone	106-68-3	Plant volatiles	Aladdin Biochemical Technology Co., LTD	Shanghai, China	98.0 %	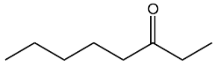
1-Octen-3-one	4312-99-6	Plant volatiles	Aladdin Biochemical Technology Co., LTD	Shanghai, China	95.0 %	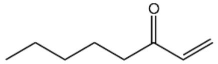
3-Octanol	589-98-0	Plant volatiles	Energy Chemical Technology Co., LTD	Shanghai, China	98.0 %	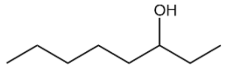
2-Methyl butyric acid	116-53-0	Plant volatiles	Aladdin Biochemical Technology Co., LTD	Shanghai, China	98.0 %	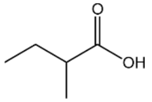
α-Pinene	2437-95-8	Plant volatiles	Sigma-Aldrich LLC.	MO, USA	98.0 %	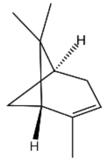
Camphene	79-92-5	Plant volatiles	Aladdin Biochemical Technology Co., LTD	Shanghai, China	98.0 %	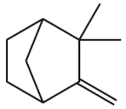

Compound	Cas No.	Source	Company	Country	Purity	Structure
Linalool oxide	60047-17-8	Plant volatiles	Aladdin Biochemical Technology Co., LTD	Shanghai, China	98.0 %	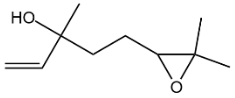
(-)-Verbenone	1196-01-6	Plant volatiles	Aladdin Biochemical Technology Co., LTD	Shanghai, China	95.0 %	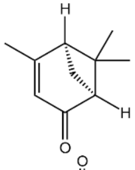
Methyl benzoate	93-58-3	Plant volatiles	Sigma-Aldrich LLC.	MO, USA	99.0 %	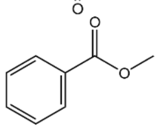
M-xylene	108-38-3	Plant volatiles	Aladdin Biochemical Technology Co., LTD	Shanghai, China	99.0 %	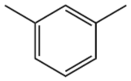
Carvone	99-49-0	Plant volatiles	Macklin Biochemical Technology Co., LTD	Shanghai, China	95.0 %	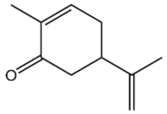
1-Octene	111-66-0	Plant volatiles	Macklin Biochemical Technology Co., LTD	Shanghai, China	98.0 %	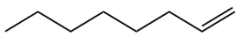
6-Methyl-5-hepten-2-one	110-93-0	Plant volatiles	Sigma-Aldrich LLC.	MO, USA	99.0 %	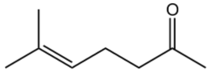
Decyl aldehyde	112-31-2	Plant volatiles	Macklin Biochemical Technology Co., LTD	Shanghai, China	97.0 %	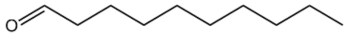
Dimethyl phthalate	131-11-3	Plant volatiles	Aladdin Biochemical Technology Co., LTD	Shanghai, China	99.0 %	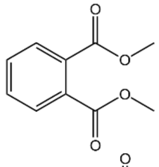
Methyl cinnamate	103-26-4	Plant volatiles	Aladdin Biochemical Technology Co., LTD	Shanghai, China	99.0 %	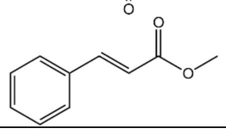

Compound	Cas No.	Source	Company	Country	Purity	Structure
Methyl anthranilate	134-20-3	Plant volatiles	Aladdin Biochemical Technology Co., LTD	Shanghai, China	99.0%	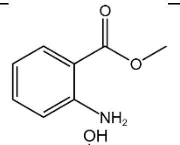
α-Terpineol	10482-56-1	Plant volatiles	Aladdin Biochemical Technology Co., LTD	Shanghai, China	98.0%	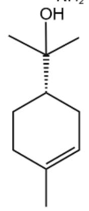
(E)-β-farnesene	18794-84-8	Plant volatiles	Aladdin Biochemical Technology Co., LTD	Shanghai, China	90.0%	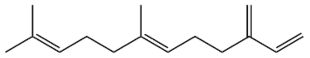
Nerolidol	7212-44-4	Plant volatiles	Sigma-Aldrich LLC.	MO, USA	98.0%	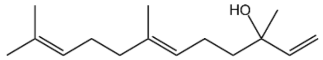
Isoeugenol	97-54-1	Plant volatiles	Aladdin Biochemical Technology Co., LTD	Shanghai, China	97.0%	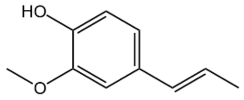
Benzyl benzoate	120-51-4	Plant volatiles	Aladdin Biochemical Technology Co., LTD	Shanghai, China	99.0%	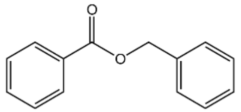
2-Hexanol	626-93-7	Plant volatiles	Aladdin Biochemical Technology Co., LTD	Shanghai, China	98.0%	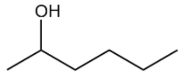
Trans-3-hexen-1-ol	544-12-7	Plant volatiles	Bidepharm Medical Technology Co., LTD	Shanghai, China	98.0%	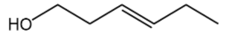
γ-Decalactone	706-14-9	Plant volatiles	Aladdin Biochemical Technology Co., LTD	Shanghai, China	98.0%	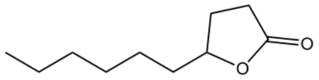
Citronellyl acetate	150-84-5	Plant volatiles	Macklin Biochemical Technology Co., LTD	Shanghai, China	96.0%	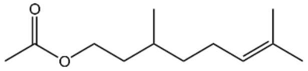

Compound	Cas No.	Source	Company	Country	Purity	Structure
(+)-Aromadendrene	489-39-4	Plant volatiles	Sigma-Aldrich LLC.	MO, USA	97.0 %	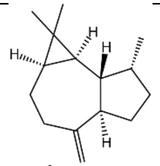
Rose oxide	16409-43-1	Plant volatiles	Heowns Chemical TecHhnology Co., LTD	Wuhan, China	96.0 %	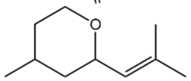
2-Tridecanone	593-08-8	Plant volatiles	Rhawn Biotechnology Co., LTD	Shanghai, China	98.0 %	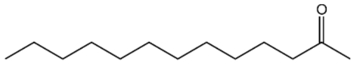
Benzoylformic acid	611-73-4	Plant volatiles	Aladdin Biochemical Technology Co., LTD	Shanghai, China	95.0 %	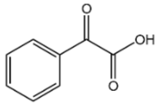
Methyl anisate	121-98-2	Plant volatiles	Aladdin Biochemical Technology Co., LTD	Shanghai, China	99.0 %	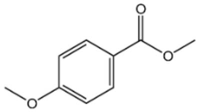
Ethyl 4-methoxybenzoate	94-30-4	Plant volatiles	Aladdin Biochemical Technology Co., LTD	Shanghai, China	98.0 %	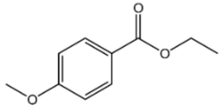
α-Phellandrene	99-83-2	Plant volatiles	Aladdin Biochemical Technology Co., LTD	Shanghai, China	99.0 %	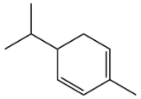
Methyl acetate	79-20-9	Plant volatiles	Aladdin Biochemical Technology Co., LTD	Shanghai, China	98.0 %	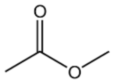
Methyl decanoate	110-42-9	Plant volatiles	Aladdin Biochemical Technology Co., LTD	Shanghai, China	98.0 %	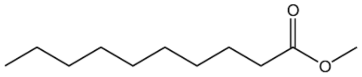
α-Ionol	25312-34-9	Plant volatiles	Aladdin Biochemical Technology Co., LTD	Shanghai, China	90.0 %	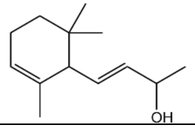

Compound	Cas No.	Source	Company	Country	Purity	Structure
Ethyl caprate	110-38-3	Plant volatiles	Aladdin Biochemical Technology Co., LTD	Shanghai, China	99.0 %	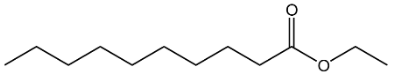
Pentamethylbenzene	700-12-9	Plant volatiles	Bidepharm Medical Technology Co., LTD	Shanghai, China	98.0 %	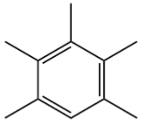
Cyclohexanone	108-94-1	Plant volatiles	Aladdin Biochemical Technology Co., LTD	Shanghai, China	99.0 %	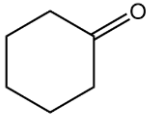
p-Tolualdehyde	104-87-0	Plant volatiles	Macklin Biochemical Technology Co., LTD	Shanghai, China	97.0 %	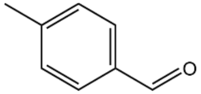
Methyl eugenol	93-15-2	Plant volatiles	Rhawn Biotechnology Co., LTD	Shanghai, China	98.0 %	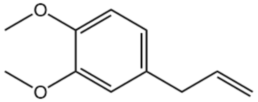
trans-2-Hexenyl acetate	2497-18-9	Plant volatiles	Sigma-Aldrich LLC.	MO, USA	98.0 %	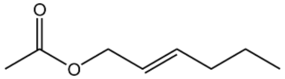
3,5-Dimethoxytoluene	4179-19-5	Plant volatiles	Bidepharm Medical Technology Co., LTD	Shanghai, China	99.9 %	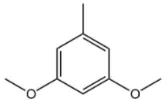
Methyl jasmonate	1211-29-6	Plant volatiles	Sigma-Aldrich LLC.	MO, USA	95.0 %	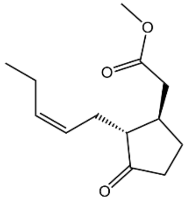
1,4-Dimethoxybenzene	150-78-7	Plant volatiles	Aladdin Biochemical Technology Co., LTD	Shanghai, China	99.0 %	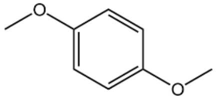
Ethyl benzoate	93-89-0	Plant volatiles	Rhawn Biotechnology Co., LTD	Shanghai, China	99.5 %	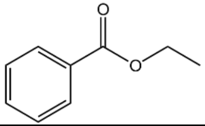

Compound	Cas No.	Source	Company	Country	Purity	Structure
Bornyl acetate	76-49-3	Plant volatiles	Macklin Biochemical Technology Co., LTD	Shanghai, China	97.0%	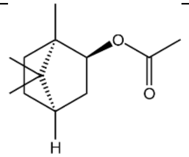
Vanillin	121-33-5	Plant volatiles	Aladdin Biochemical Technology Co., LTD	Shanghai, China	99.0%	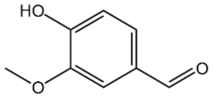
α-Terpinene	99-86-5	Plant volatiles	Aladdin Biochemical Technology Co., LTD	Shanghai, China	90.0%	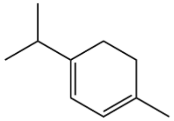
Acetophenone	98-86-2	Plant volatiles	Aladdin Biochemical Technology Co., LTD	Shanghai, China	99.5%	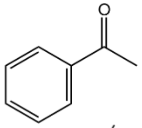
Isophorone	78-59-1	Plant volatiles	Aladdin Biochemical Technology Co., LTD	Shanghai, China	97.0%	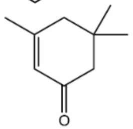
p-Anisaldehyde	123-11-5	Plant volatiles	Aladdin Biochemical Technology Co., LTD	Shanghai, China	98.0%	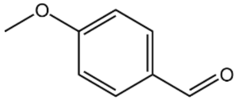
trans-Anethole	4180-23-8	Plant volatiles	Aladdin Biochemical Technology Co., LTD	Shanghai, China	99.0%	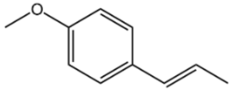
cis-Anethole	104-46-1	Plant volatiles	Aladdin Biochemical Technology Co., LTD	Shanghai, China	99.8%	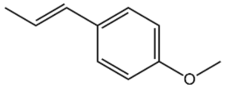
p-Cymene	99-87-6	Plant volatiles	Macklin Biochemical Technology Co., LTD	Shanghai, China	98.0%	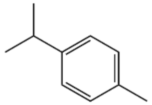
(E,E)-Farnesol	106-28-5	Plant volatiles	Aladdin Biochemical Technology Co., LTD	Shanghai, China	97.6%	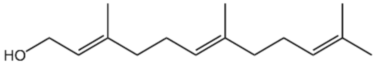

Compound	Cas No.	Source	Company	Country	Purity	Structure
(E,E)-α-Farnesene	502-61-4	Plant volatiles	Sigma-Aldrich LLC.	MO, USA	98.0 %	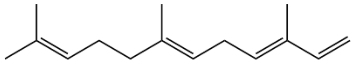
4-Allylanisole	140-67-0	Plant volatiles	Aladdin Biochemical Technology Co., LTD	Shanghai, China	97.0 %	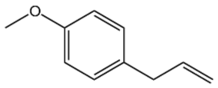
4-Oxoisophorone	1125-21-9	Plant volatiles	Energy Chemical Technology Co., LTD	Shanghai, China	98.0 %	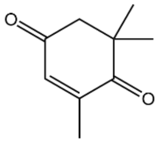
2-Aminobenzaldehyde	529-23-7	Plant volatiles	Bidepharm Medical Technology Co., LTD	Shanghai, China	98.0 %	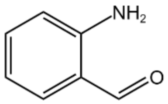
3,7-Dimethylocta-1,6-dien-3-yl acetate	115-95-7	Plant volatiles	Bidepharm Medical Technology Co., LTD	Shanghai, China	98.0 %	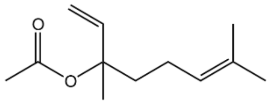
2-Methylbutyraldehyde	96-17-3	Plant volatiles	Bidepharm Medical Technology Co., LTD	Shanghai, China	95.0 %	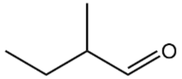
1,4-Xylene	106-42-3	Plant volatiles	Heowns Chemical TecHhnology Co., LTD	Wuhan, China	98.0 %	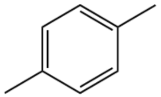
Menthone	10458-14-7	Plant volatiles	Bidepharm Medical Technology Co., LTD	Shanghai, China	98.0 %	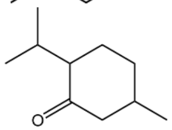
2-Nonanone	821-55-6	Plant volatiles	Aladdin Biochemical Technology Co., LTD	Shanghai, China	99.0 %	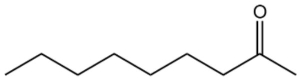
γ-Octanoic lactone	104-50-7	Plant volatiles	Bidepharm Medical Technology Co., LTD	Shanghai, China	98.0 %	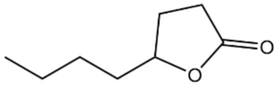

Compound	Cas No.	Source	Company	Country	Purity	Structure
Ethyl 2-hydroxybenzoate	118-61-6	Plant volatiles	Bidepharm Medical Technology Co., LTD	Shanghai, China	99.8%	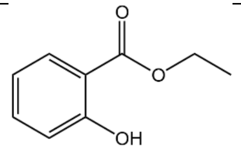
Isovaleraldehyde	590-86-3	Plant volatiles	Aladdin Biochemical Technology Co., LTD	Shanghai, China	98.0%	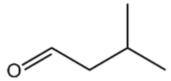
1-Decene	872-05-9	Plant volatiles	Aladdin Biochemical Technology Co., LTD	Shanghai, China	95.0%	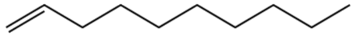
2-Pentadecanone	2345-28-0	Plant volatiles	Sigma-Aldrich LLC.	MO, USA	98.5%	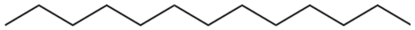

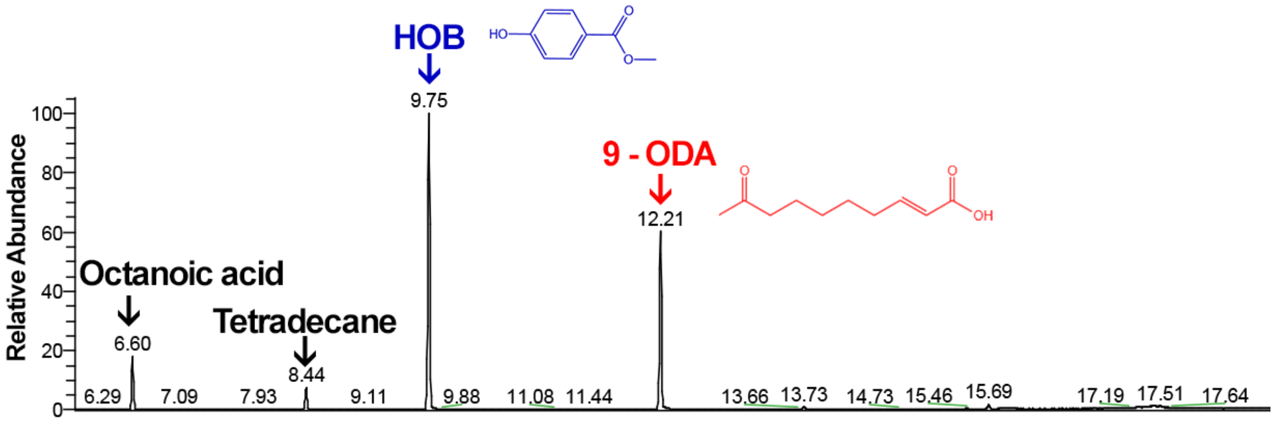

Supplementary Fig. 1 Chromatogram of synthetic 9-ODA and HOB standards.

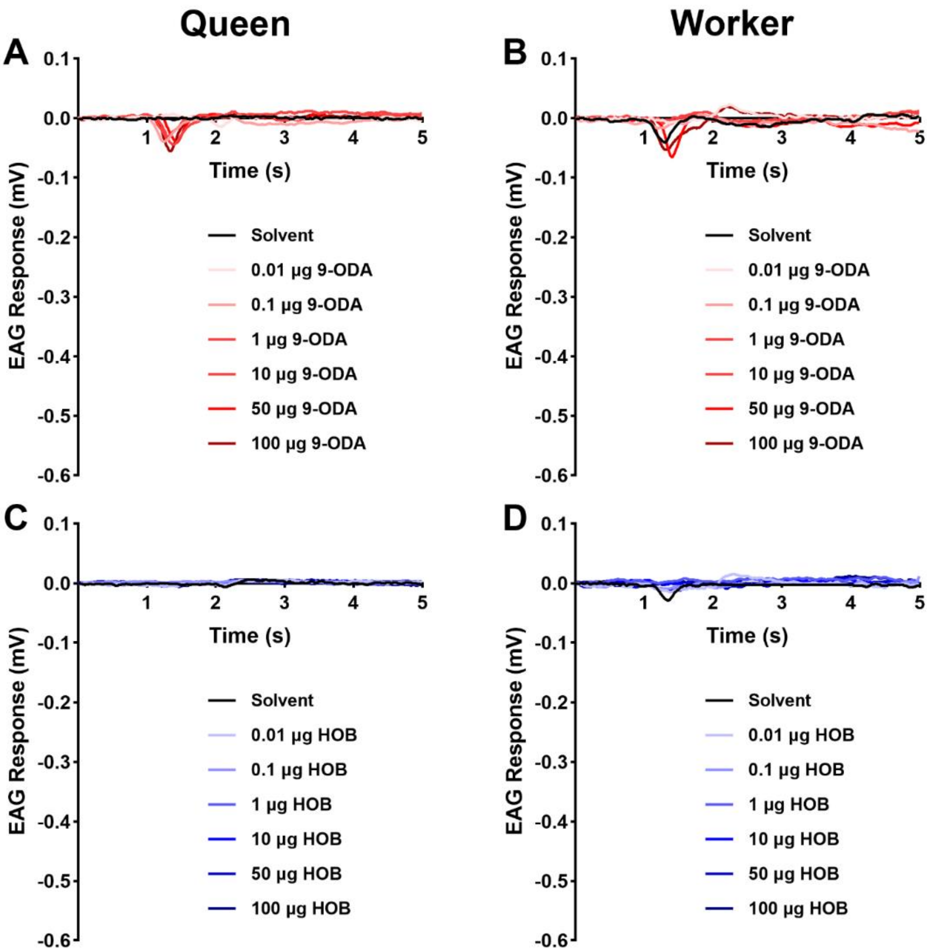

Supplementary Fig. 2 EAG traces of queens and workers in response to 9-ODA and HOB.

Responses of queens and workers elicited by the increasing doses (0.01 to 100 µg) of (A, B) 9-ODA and (C, D) HOB.

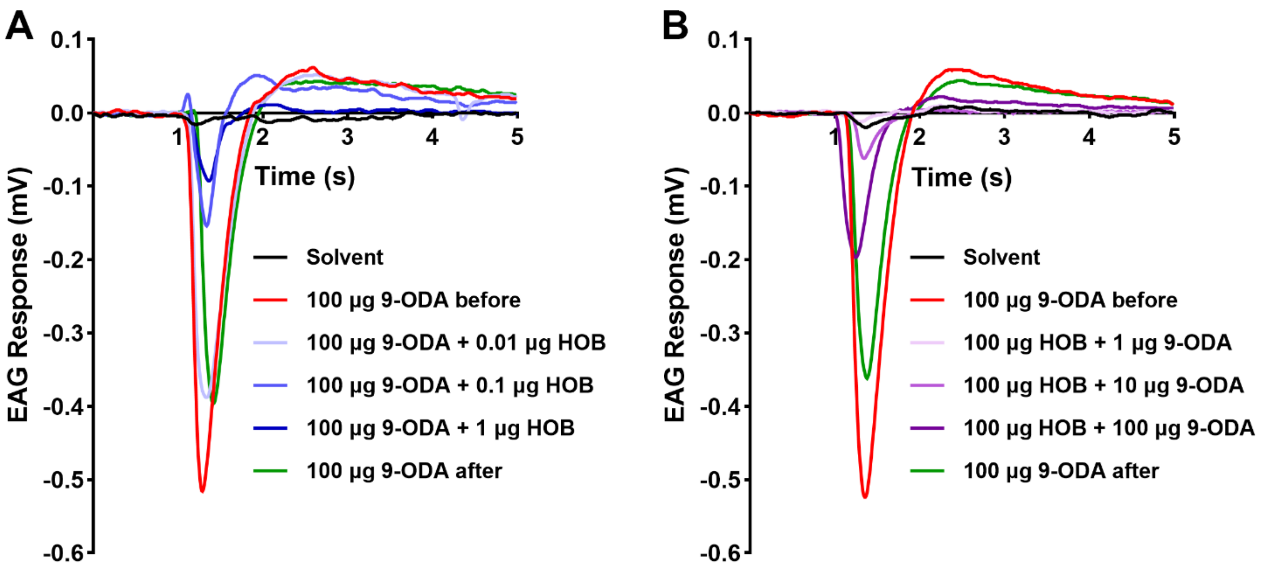

Supplementary Fig. 3 Representative EAG inhibitory trace of HOB to 9-ODA.

EAG responses of (A) 100 µg 9-ODA with 0.01 to 1 µg HOB and (B) 100 µg HOB with 1 to 100 µg 9-ODA.

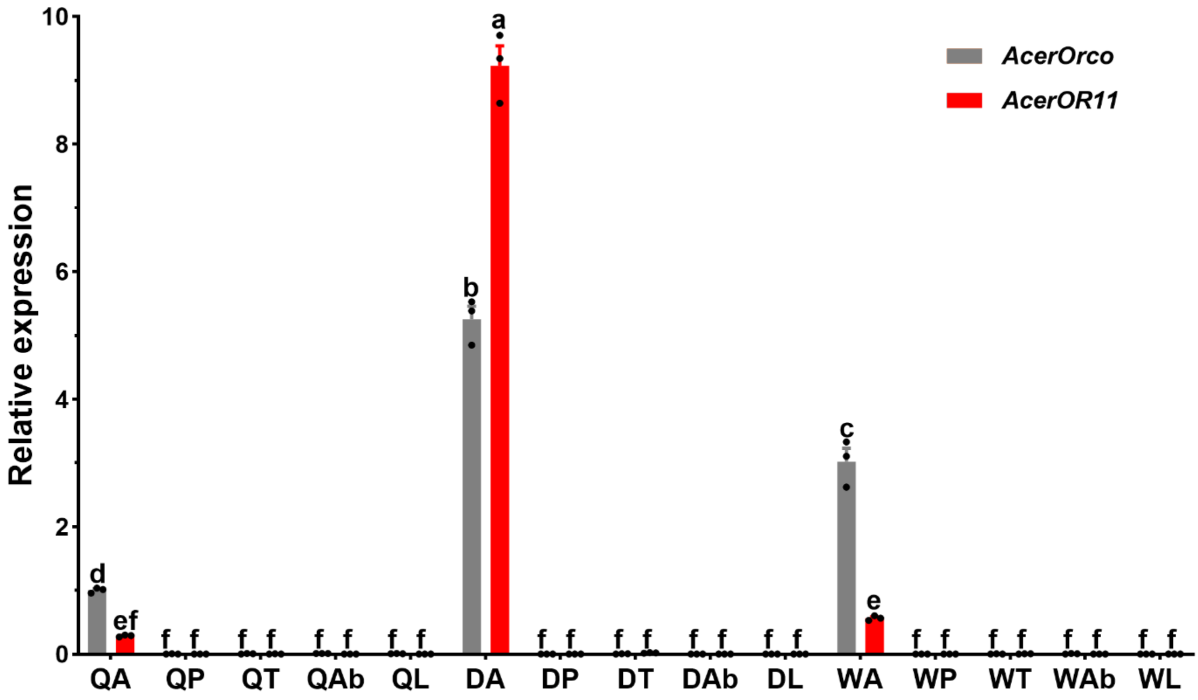

Supplementary Fig. 4 RT-qPCR results of *AcerOr11* and *AcerOrco* in *A. cerana*.

QA: Queen antenna; QP: Queen proboscis; QT: Queen thorax; QAb: Queen abdomen; QL: Queen legs; DA: Drone antenna; DP: Drone proboscis; DT: Drone thorax; DAb: Drone abdomen; DL: Drone legs; WA: Worker antenna; WP: Worker proboscis; WT: Worker thorax; WAb: Worker abdomen; WL: Worker legs. Different lowercase letters indicate significant differences based on a one-way ANOVA followed by Tukey's multiple comparison test ($P < 0.05$, N=3). Data are the mean ± standard error.

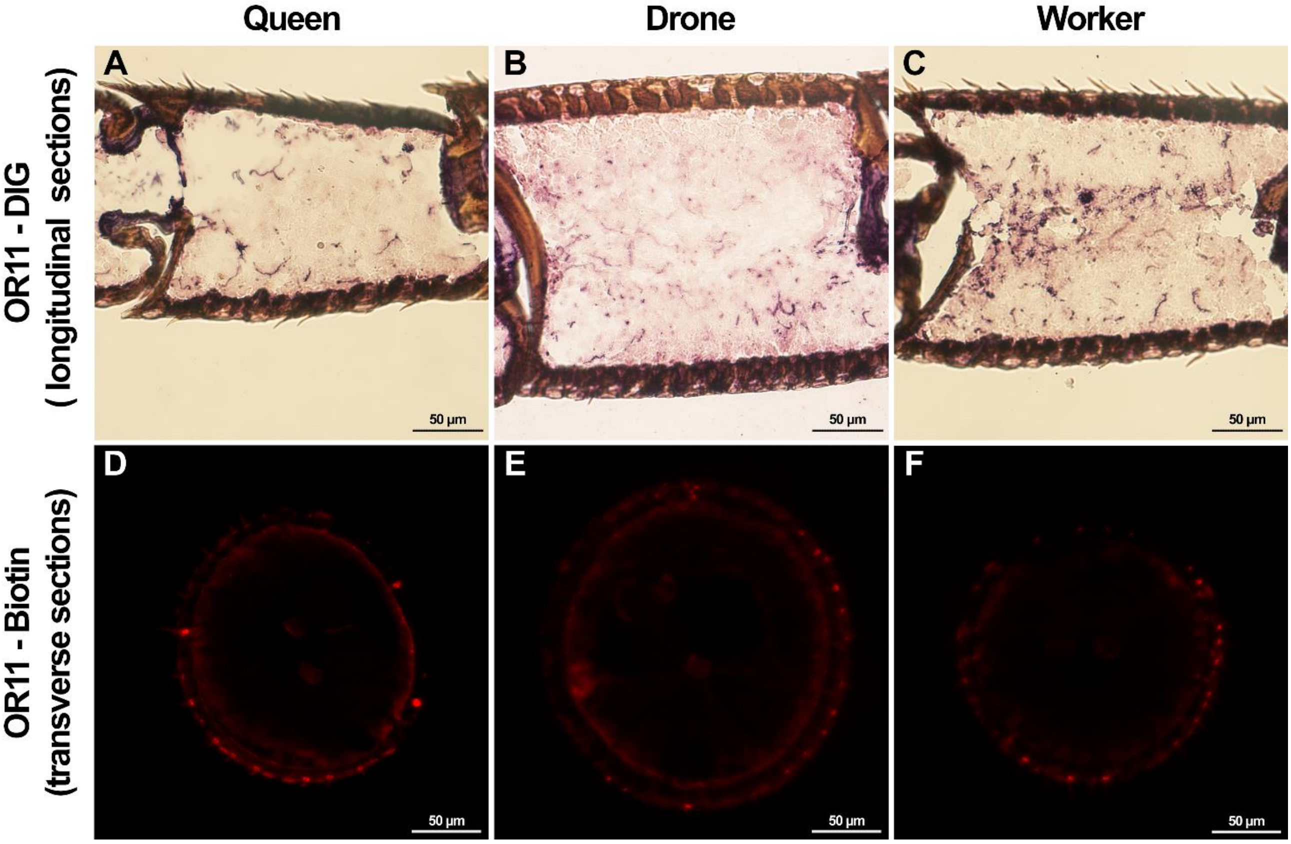

Supplementary Fig. 5 Sense RNA probe of FISH of AcerOr11 from the antennae of *A. cerana*.

(A-C) In situ hybridization with the sense Or11-DIG probe on longitudinal sections. (D-F) Fluorescence in situ hybridization with the sense Or11-Biotin specific probe on longitudinal sections.

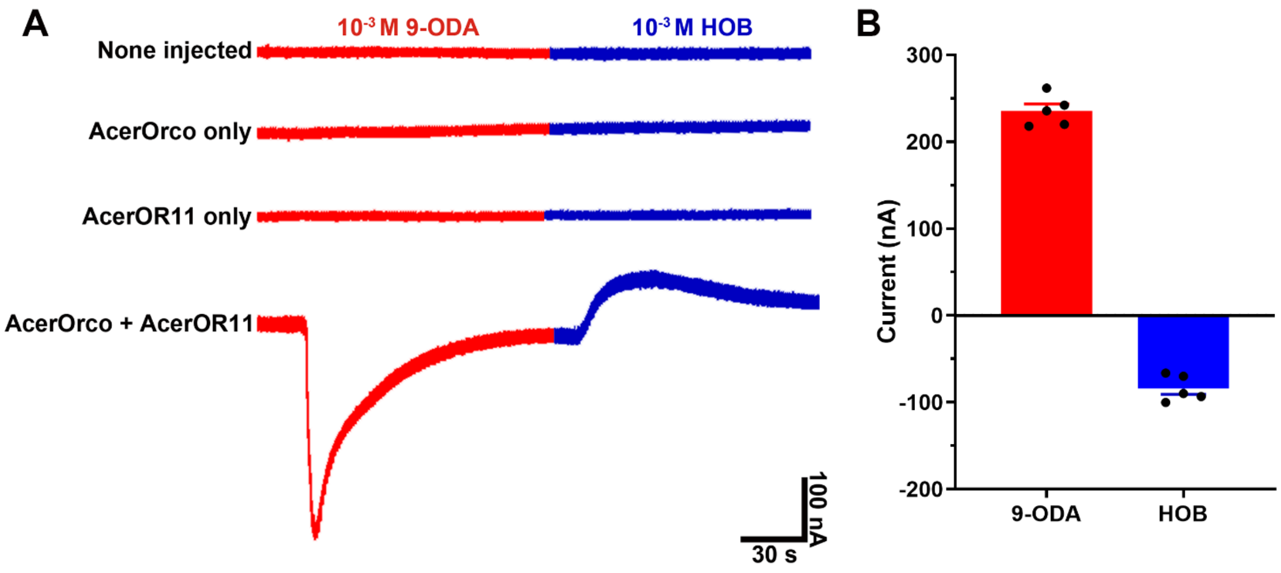

Supplementary Fig. 6 Representative TEVC trace of 9-ODA and HOB.

(A) Control experiments with non-injected oocytes and oocytes expressing AcerOrco, AcerOr11, or AcerOr11/AcerOrco. (B) AcerOr11/AcerOrco expressing oocytes response to 9-ODA and HOB at 10^{-3} M concentration (N = 5).

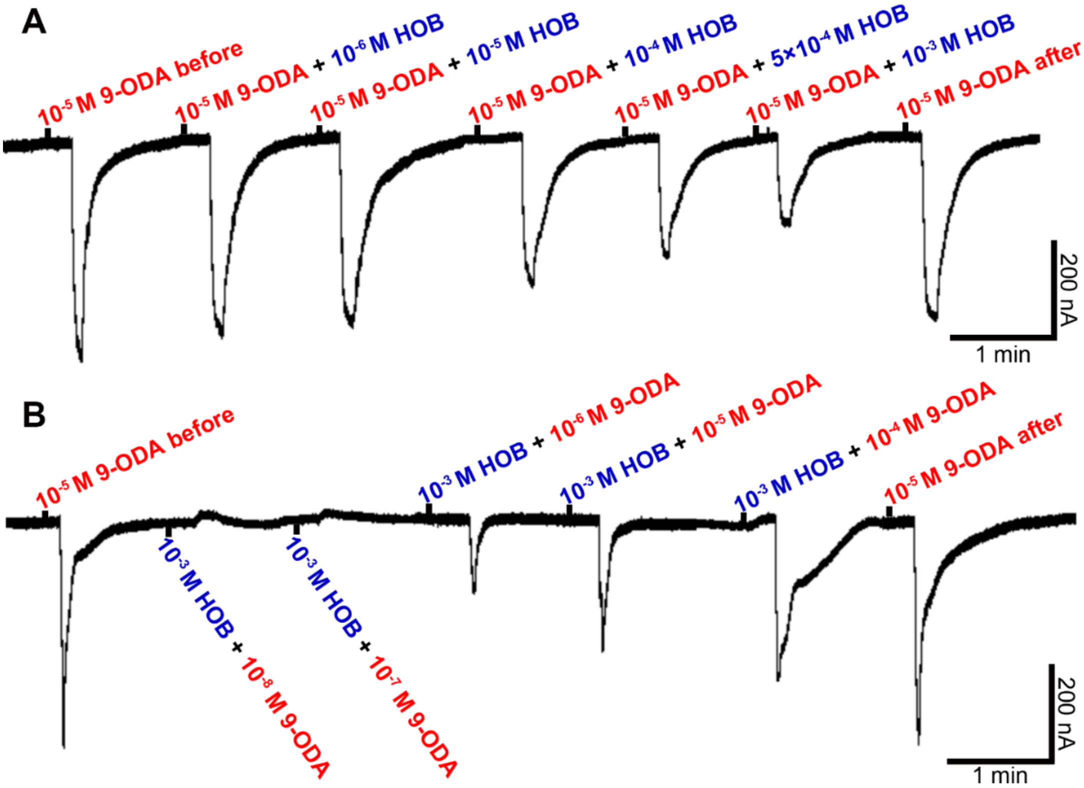

Supplementary Fig. 7 Inhibitory trace of HOB to 9-ODA in *Xenopus* oocytes.

(A) TEVC response of AcerOR11 to 10^{-5} M 9-ODA with 10^{-6} to 10^{-3} M HOB. (B) TEVC response of AcerOr11 to 10^{-3} M HOB with 10^{-8} to 10^{-4} M 9-ODA.
